# Identifying research practices toward achieving health equity principles within the Cancer Prevention and Control Research Network

**DOI:** 10.1007/s10552-023-01674-2

**Published:** 2023-02-24

**Authors:** Prajakta Adsul, Jessica Islam, Perla Chebli, Julie Kranick, Sarah Nash, Hannah Arem, Stephanie Wheeler, Melissa Lopez-Pentecost, Victoria Foster, Rashmi K. Sharma, Tisha Felder, Betsy Risendal, Enmanuel A. Chavarria, Simona Kwon, Rachel Hirschey, Chau Trinh-Shevrin

**Affiliations:** 1 Department of Internal Medicine, University of New Mexico, Albuquerque, NM USA; 2 Cancer Control and Populations Sciences Research Program, Cancer Research Facility (CRF), University of New Mexico Comprehensive Cancer Center, Albuquerque, NM USA; 3 Moffitt Cancer Center, Tampa, FL USA; 4 Department of Population Health, NYU Grossman School of Medicine, Section for Health Equity, New York, NY USA; 5 Department of Epidemiology, College of Public Health, University of Iowa, Iowa City, IA USA; 6 MedStar Health Research Institute, Washington, DC USA; 7 Department of Health Policy and Management, University of North Carolina at Chapel Hill, Chapel Hill, NC USA; 8 University of Miami School of Medicine, Miami, FL USA; 9 Division of General Internal Medicine, University of Washington School of Medicine, Seattle, WA USA; 10 University of South Carolina, Columbia, SC USA; 11 Colorado School of Public Health, University of Colorado Cancer Center, Aurora, CO USA; 12 Department of Behavioral, Social, and Health Education Sciences, Emory University, Rollins School of Public Health, Atlanta, GA USA; 13 School of Nursing, University of North Carolina at Chapel Hill, Chapel Hill, NC USA

**Keywords:** Cancer prevention and control, Health equity, Toolkit, Research

## Abstract

**Purpose:**

Although there is national recognition for health equity-oriented research, there is limited guidance for researchers to engage partnerships that promote health equity in cancer research. The Cancer Prevention and Control Research Network’s (CPCRN) Health Equity Work Group developed a toolkit to guide researchers in equitable collaborations.

**Methods:**

The CPCRN’s Health Equity Work Group collectively outlined health and racial equity principles guiding research collaborations with partners that include communities, community-based organizations, implementing partners in the clinical setting including providers and health care organizations, and policy makers. Using a network-wide survey to crowdsource information around ongoing practices, we leveraged and integrated the network’s experience and collaborations.

**Results:**

Data from the survey formed the preliminary basis for the toolkit, with a focus on sharing fiscal resources with partners, training and capacity building, collaborative decision-making, community-driven research agenda setting, and sustainability. The final toolkit provides reflection considerations for researchers and collated exemplary resources, supported by the contemporary research.

**Conclusions:**

The toolkit provides a guide to researchers at all experience levels wanting to engage in equitable research collaborations. Future efforts are underway to evaluate whether and how researchers within and outside CPCRN are able to incorporate these principles in research collaborations.

**Supplementary Information:**

The online version contains supplementary material available at 10.1007/s10552-023-01674-2.

## Introduction

Recent racial and social justice movements and the disproportionate impact of COVID-19 on many historically marginalized communities in the United States have further highlighted the urgency of addressing health and racial inequities. Many scientific communities, including those focused on public health [1], healthcare [2], cancer [3], and medicine [4], have clarified or re-invigorated their commitment to health equity. Health equity as defined by Paula Braveman [5] refers to, “the highest possible standard of health for all people and giving special attention to the needs of those at greatest risk of poor health, based on social conditions.”

National organizations such as the Centers for Disease Control and Prevention (CDC) and the National Academies of Science, Engineering, and Medicine (NASEM) have developed accessible tools for communities and public health practitioners encompassing community and practice-based strategies and resources for advancing health equity. For example, the CDC’s Practitioner Guide for Advancing Health Equity outlines a set of foundational skills for public health practitioners to incorporate efforts toward advancing health equity into the field of public health, including capacity building, meaningful community engagement, developing partnerships and coalitions, understanding health inequities, strategy selection, design, and implementation, and evaluation efforts [6]. In 2017, NASEM published a report spotlighting a set of community-based solutions to address root causes of health inequities such as education, transportation, housing, planning, public health, among others [7]. Specifically, the NASEM report provides examples of solutions implemented by communities, such as promoting community-based approaches and health equity through civil rights legislation and utilizing health impact assessments, and a curated list of tools with the purpose of disseminating such efforts for wider adoption by other communities to foster change in their own unique environments.

While available tools serve as guides for practitioners and others serving our communities, there remains a gap for investigators and their teams who are new to health-equity-oriented research, especially those that are working in the field of cancer prevention and control [8]. This gap is further exacerbated in the unprecedented number of investigators that have shifted their attention toward health equity, often lacking previous experience, necessary skills, or sustained commitment to communities experiencing disparities. Referred to as “health equity tourism” by Lett and colleagues, such a deficit in skills and commitment can lead to unintended consequence of causing more harm than benefit to the communities [9]. To engage in health equity-oriented research, investigators must shift from traditional research methods to a paradigm that incorporates equitable practices and processes within research collaborations. Such a shift requires thoughtful planning and operationalization of key strategies needed to advance health equity meaningfully and sustainably.

## Cancer Prevention and Control Research Network's commitment to health equity

Within the field of cancer prevention and control, identifying health and racial equity goals is necessary because of slow progress toward addressing cancer inequities that are vast and may be widening [10]. As a “thematic research network” of the CDC’s Prevention Research Centers, the Cancer Prevention and Control Research Network (CPCRN) comprises academic, public health, and community partners; all committed and working toward health equity in many of the research efforts. While health and racial equity have been the cornerstone of many of its research efforts, the CPCRN, just recently, has explicitly operationalized a network-wide equity mission into its charter. Described by Chebli, et al. [11], in response to the growing need to address equity, the CPCRN Health Equity Workgroup led a collaborative effort with CPCRN members to define a set of core health and racial equity principles that reflect and guide the ongoing and future research efforts of the network.

As described in-depth elsewhere [11], this process was initially informed by a high-level review of the literature from which the preliminary equity principles were identified. When identifying the principles, it was of paramount importance to the Health Equity Workgroup to ensure robust participation from within the CPCRN in reviewing and vetting the principles. These principles were then followed by a cross-center survey, which asked respondents to rate the applicability of each principle, suggest additional principles, provide illustrative examples from their work, and propose relevant measures (described in the following sections). Survey finding highlights are described in Chebli, et al. [11], and provided a foundation of operational examples from each CPCRN center and were the basis for committee discussions and consensus building activities across the network to refine and finalize principles. Through these network-wide activities, in which each network center actively participated, the Health Equity Workgroup strengthened support for the CPCRN focus on health equity. The health and racial equity principles are described in Chebli, et al. [11], and are presented in Table 1.

**Table 1:** CPCRN health & racial equity principles

No	Principle
1.	Engage in power-sharing and capacity building with partners
2.	Address community priorities through community engagement and co-creation of research
3.	Explore and address the systems and structural root causes of cancer disparities
4.	Build a system of accountability between research and community partners
5.	Establish transparent relationships with community partners
6.	Prioritize the sustainability of research benefits for community partners
7.	Center racial equity in cancer prevention and control research
8.	Engage in equitable data collection, analysis, interpretation, and dissemination practices
9.	Integrate knowledge translation, implementation, and dissemination into research plans

The purpose of our current effort was to address the gap in terms of guidelines for researchers through the development and dissemination of a toolkit titled, “Equitable Research Collaborations: A toolkit for researchers based on the Cancer Prevention and Control Research Network (CPCRN) principles for Health Equity.” We provide a set of considerations on how the CPCRN’s Health Equity Principles can be operationalized; examples of actions that have been undertaken by investigators at various CPCRN sites; questions for researchers to reflect on when incorporating these principles; and resources and methods for their assessment.

## Practices undertaken by CPCRN members and proposed measures

As a first step toward this purpose, we conducted a CPCRN-wide survey, in which the participants were asked to provide illustrative examples from their work, either through published papers or through a narrative description of practices adopted in ongoing research projects. Survey data were extracted to conduct a thematic analysis [12] which resulted in broad themes describing areas wherein CPCRN members are actively integrating these principles into ongoing and proposed research projects. We analyzed data from 28 members representing all current CPCRN sites, and uncovered five overarching themes that collectively categorized the practices undertaken by CPCRN investigators in their research projects to implement the CPCRN health and racial equity principles.

These five overarching themes were as follows: (1) *Fiscal*: this theme highlighted practices undertaken by the CPCRN member in fairly compensating community partners; and, offering mini-grant opportunities; (2) *Training*: this theme was geared toward co-leading training programs with community partners to build their capacity for research engagement; (3) *Collaborative decision making*: this theme reflected the establishing of Community Advisory Boards that could guide research decisions; deciding with community partners at each step of the research who needs to be involved and how; and, conducting regular meetings with both researchers and community members filling established roles; (4) *Community-driven agenda setting*: this theme included conducting community needs assessments and aligning projects with community defined needs; responding to community partners’ requests for support; distributing research outcomes across racial and ethnic and, urban/rural populations; generating community-based participatory research-driven solutions to disparities; and, formalizing plans to incorporate health equity; and (5) *Sustainability*: this theme reflected grounding interventions in current systems or practice-based research; identifying sources of support for services; and utilizing components of the intervention that can continue beyond the funding period. Collectively, these themes informed the development of the toolkit described below.

Survey respondents were also asked to provide evaluation measures they may have used in ongoing or proposed projects. Notably, we received very few responses to this prompt, suggesting a limited understanding and capacity around how to incorporate these principles. The survey responses confirmed a growing recognition of the limited guidance for making progress on achieving these principles in practice. Solidifying our commitment to health and racial equity, promote accountability, and facilitate implementation, the Health Equity Workgroup developed a toolkit of resources, designed to aid engagement with the principles through reflection questions, and guide current and/or future work in the implementation of the principles through operational examples and recommended methods for assessment along with additional suggested resources.

## Development of the toolkit–equitable research collaborations

Building on the survey data and complementary to the formalization of the health and racial equity principles, the Health Equity Workgroup identified other deliverables—including this toolkit—that could be useful for researchers as they engage with communities to address health equity. In the Health Equity Workgroup discussions, there were several conversations around the idea of a checklist that allows investigators to examine whether or not the proposed or ongoing research incorporates these health and racial equity principles. In collective reflection, we were cognizant of the fact that restrictions on funding and research topic at hand may guide where the research team may fall on such a checklist. Instead of an evaluative tool, we believed in the need for a reflective toolkit.

The toolkit development process was iterative: between June 2021 and July 2022, the Health Equity Workgroup met bi-weekly to discuss the structure and content of the toolkit. All members of the Health Equity workgroup who were involved in the development of the toolkit had experience conducting community-engaged, health equity-oriented, cancer prevention, and control research. Where additional expertise was needed to support the toolkit’s development, expertise was sought from within the CPCRN or its affiliates. Each principle was randomly assigned to at least two Health Equity Workgroup members; each pair then built out the resources under their assigned principles. Informed by results from the CPCRN survey described above, Health Equity Workgroup members identified strategies that CPCRN members were already implementing to apply the equity principles. Our goal was to provide researchers with feasible examples that they could use in their own work. At each of our bi-weekly meetings, the Health Equity Workgroup collaboratively reviewed, discussed, and refined each principle’s section. Thus, each member of the Health Equity Workgroup had multiple opportunities to review each section of the toolkit throughout its development. Once the draft toolkit had been completed, it was then shared with CPCRN members and affiliates for review and feedback. Input was collated by Health Equity Workgroup members and incorporated into the final draft of the toolkit.

We have provided the most recent version of the toolkit as an Online Resource (Online Resource 1) and described the contents briefly in this manuscript. Our goal is to continuously update the toolkit to include feedback from users. In this spirit, we invite the readers of this manuscript and toolkit to contribute their thoughts for improvement, including the suggestion of additional tools, measures, evaluation methods, and reflections, either by contacting the corresponding author for this manuscript or on the contact information provided for the toolkit.

Briefly, the toolkit is structured as follows: there are nine sections organized according to each CPCRN equity principle. The section for each principle consists of four sub-sections: (1) operationalizing the principle for research collaboration; (2) practices undertaken or are in-progress in CPCRN projects; (3) reflection questions to guide researchers; and (4) resources and methods for assessment. The content under the found sub-sections is provided in Table 2.

**Table 2:** Structure of the toolkit

No	Section title	Content and examples
1	Operationalizing the principle for research collaboration	The first sub-section defines how each principle can be operationalized for research collaborations. This sub-section is not an exhaustive list; rather, it is designed to get researchers thinking about different ways in which these principles could be put into action in research projects, especially projects that include a community-engaged component
2	Practices undertaken or in-progress in CPCRN projects	The second sub-section provides examples of how the principle has been operationalized in existing Cancer Prevention and Control Research Network (CPCRN) projects. These examples come directly from the CPCRN investigators through data provided during the survey or input from investigators and affiliates working across the research network. This sub-section is designed to help researchers contemplate feasible actions to implement within their own research
3	Reflection questions to guide researchers	The third sub-section comprises reflection questions which are designed to be thought-provoking prompts to help researchers begin the process of engaging with each principle For example, under Principle 1 (Engage in power-sharing and capacity building with partners), one question is “How are partnership power dynamics defined, acknowledged, and addressed?” In another example, for Principle 7 (Center racial equity in cancer prevention and control), another question asks the researcher and their teams to consider, “Is your research team trained in understanding the nuances of unconscious bias and how it influences the way they engage with study participants or community partners?” Researchers can use these questions to reflect on their ongoing work, and consider areas where they could make modifications to improve health equity
4	Resources and methods for assessment	In the fourth sub-section, we present a list of resources that researchers might find useful to support each principle. This section was primarily curated using the contemporary literature as well as input from CPCRN-wide researchers working in the spaces of cancer prevention and control and health equity In this sub-section, we have included short annotations to orient researchers to these resources and help them identify which might be most relevant to their ongoing work or reflective process

**Fig. 1:**
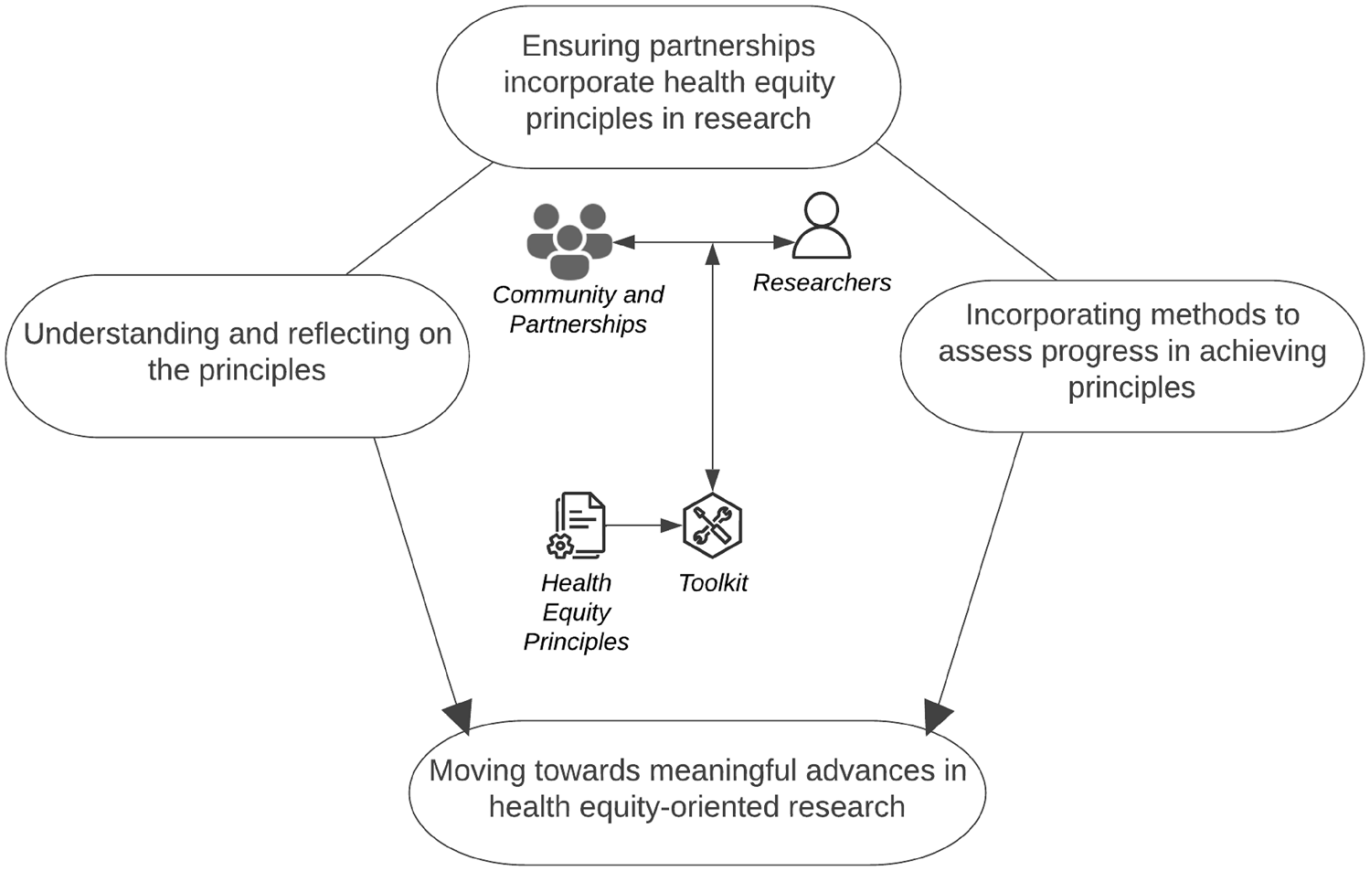
Incorporating health equity principles in cancer prevention and control research

We intend for this toolkit to be dynamic and iterative, and the list of resources to be revised and updated periodically. A visual depicting the potential use of this toolkit is provided in Fig. 1. Similar to a builder’s, engineer’s, or craftsperson’s toolkit composed of various types of equipment to implement in achieving projects or tasks of their trade, we developed a toolkit that was accessible, user-friendly, and could be leveraged by researchers of all skill levels as they work toward health and racial equity.

## Strengths and limitations

While we appreciate the strengths of the inaugural toolkit, we also recognize several limitations. First, the toolkit was developed by CPCRN investigators with distinguished experience and training in health equity research, yet perspectives of health equity researchers who are not part of the CPCRN network remain to be drawn upon and included in future efforts. We began to address this limitation by including resources outside the scope of CPCRN’s body of work. We extensively referenced literature and diligently included contemporary resources from health equity researchers both, within the CPCRN network and beyond. Although we include operational examples from CPCRN investigators, future efforts are currently directed toward supplementing these examples with more comprehensive case studies. Second, while the toolkit provides guidance and recommendations, it does not offer evaluative measures to assess whether or how well researchers are applying the equity principles. We included reflection questions in the toolkit to assist researchers in self-evaluation of their processes and research. Understandably, future efforts are also ongoing to develop evaluative measures that assess to what extent and how well equity principles are incorporated in research. Finally, we also recognize that future efforts for refining both the principles and accompanying toolkit should reflect broader perspectives and include the voices of community members that have been involved in the research endeavors. The current inaugural effort commences a sound foundation that build’s researcher’s capacity to begin the work of engaging with diverse communities. The current toolkit content was developed by community-engaged researchers drawing from lessons learned while conducting research for improved health outcomes alongside and with the communities served. Active efforts for a forthcoming next iteration are underway to further enhance the toolkit and incorporate CPCRN community partners’ input.

### Implications and future directions

Health developments, technology, global pandemic, and social unrest in recent years have highlighted the need for health equity-focused research in cancer prevention and control.

Toolkits have been considered an important strategy to promote innovation and implementation [13]; specifically, the implementation science literature has acknowledged the use of a toolkit as an important implementation strategy for improving healthcare [14]. The CPCRN Health Equity Workgroup developed a reflective toolkit to guide researchers in the development of community-engaged health equity research. This toolkit is dynamic and as such is a work in-progress, as is much of health equity-oriented research. Our aim is for the toolkit to serve as a starting point for researchers interested in racial and health equity work; and, as a go-to reference for the well-versed researchers already active in racial and health equity work as it provides a rigorous, literature-guided, pathway to incorporate health and racial equity principles into research. By disseminating it to investigators, especially early career scholars, within the CPCRN and beyond, we hope that researchers will find this toolkit helpful in reinforcing the principles in research grants, publications, and principles. In future initiatives, we hope to center the toolkit in community and partner voices and focus on developing tools that can help assess progress and impact of application of the health equity principles across the network.

### Supplementary Information

Below is the link to the electronic supplementary material.

Supplementary file1 (PDF 942 KB)

## Data Availability

All datasets generated during the current study can be made available from the corresponding author on a reasonable request.
