# Supplementary material for: Identifying research practices toward achieving health equity principles within the Cancer Prevention and Control Research Network
Source: Cancer Causes Control. 2023 Feb 24;34(Suppl 1):15–21. doi: 10.1007/s10552-023-01674-2 (PMC9950692; doi:10.1007/s10552-023-01674-2)
Supplement: Supplementary file 1 — Supplementary file1 (PDF 942 KB) [file 10552_2023_1674_MOESM1_ESM.pdf]

Sept 20, 2022

CPCRN
Cancer Prevention and Control Research Network

Equitable Research Collaborations

A toolkit for researchers based on the Cancer Prevention and Control

This toolkit is supported by the Cancer Prevention and Control Research Network (CPCRN), a thematic network of the Prevention Research Center Program and supported by the Centers for Disease Control and Prevention (CDC). Work on this toolkit was funded in part by the Division of Cancer Prevention and Control, National Center for Chronic Disease Prevention and Health Promotion of the Centers for Disease Control and Prevention, U.S. Department of Health and Human Services (HHS) under Cooperative Agreement Numbers [Colorado School of Public Health U48 DP006399, Emory University U48 DP006377, New York University School of Medicine CUNY U48 DP006396, University of Arizona U48 DP006413, University of Iowa U48 DP006389, University of North Carolina at Chapel Hill U48 DP006400, University of South Carolina U48 DP006401, University of Washington U48 DP006398]. Additional funding for this includes funding from NIMHD (K23 MD015719-01 (PI: Hirschey), and the American Cancer Society (131567-IRG-17-178-22-IRG; PIs: Ozbun, M; Pilot PI: Adsul, P). The findings and conclusions in this toolkit are those of the authors and do not necessarily represent the official views of, nor an endorsement, by CDC/HHS, or the U.S. Government.

PRC
PREVENTION RESEARCH CENTERS

CPCRN

Cancer Prevention and Control Research Network

Acknowledgements

This document was created by the Health Equity Working Group with the Cancer Prevention and Control Research Network (CPRCN). We want to specifically thank individuals listed at the end of the document that have contributed to this document.

Suggested citation

Health Equity Working Group with the Cancer Prevention and Control Research Network (CPRCN). (2022) Equitable research collaborations: A toolkit for researchers based on the Cancer Prevention and Control Research Network principles for health equity

Associated Development Manuscript

Adsul P, Islam J, Chebli P, Kranick J, Nash S, Arem H, Wheeler S, Lopez-Pentecost M, Foster V, Sharma RK, Felder T, Risendal B, Chavarria EA, Kwon S, Hirschey R, Trinh-Shevrin C. (2023). Identifying research practices towards achieving health equity principles within the Cancer Prevention and Control Research Network. Cancer Causes and Control. Corresponding author: Dr. Prajakta Adsul, University of New Mexico Comprehensive Cancer Center, [***PAdsul@salud.unm.edu***](mailto:PAdsul@salud.unm.edu)

CPCRN

Cancer Prevention and Control Research Network

Terminology

- Health Equity: “Health equity means that everyone has a fair and just opportunity to be as healthy as possible. This requires removing obstacles to health such as poverty, discrimination, and their consequences, including powerlessness and lack of access to good jobs with fair pay, quality education and housing, safe environments, and health care.” (Braveman, 2014)

- Health Disparities: “Health differences based on one or more health outcomes that adversely affects defined disadvantaged populations.” (NIMHD)

- Partners: For the purposes of this document, we consider partners to be individuals from the community, community-based organizations, implementing partners in the clinical setting including physicians and clinical staff, as well as partners that are involved in making and influencing policies.

Vision and Purpose for this document

We believe that to engage in health equity oriented research, we must shift from traditional approaches to research methodologies that incorporate equitable practices and processes within our research collaborations. As with a true toolbox, where tools may serve a multitude of implementations and projects, this toolkit is parallel for researchers at any career stage working within and beyond cancer prevention and control research.

This document is created to complement the Health and Racial Equity Principles proposed by the Cancer Prevention and Control Research Network. We build on the nine equity principles that were finalized following a multi-phase, participatory approach consisting of a literature review of existing research frameworks, a survey disseminated to members from and affiliates from CPCRN centers, and multiple rounds of consensus building.

As a group, we recognize that this document is only the starting point for reflections, best practices, and resources, to guide equitable research collaborations such that they contribute towards health equity outcomes. All suggestions and feedback are welcome as we continue to develop this work. Please email Julie Kranick (Julie.Kranick@nyulangone.org) to provide suggestions and feedback on this document.

CPCRN
Cancer Prevention and Control Research Network

Health and Racial Equity Principles

Established by the Centers for Disease Control and Prevention (CDC) and the National Cancer Institute (NCI), in 2002, the Cancer Prevention and Control Research Network (CPCRN), is a network of academic, clinical, and community partners whose goals are to accelerate the uptake of evidence-based strategies in cancer prevention and control.[1]

In a unified commitment to centering equity in our cancer prevention and control research, the CPCRN established the Health Equity (HE) Workgroup in 2020, which comprised of representatives from all eight CPCRN Collaborating Centers,[2] the Coordinating Center, funders, and affiliates. Using multiple methods including an in-depth literature review and a consensus building exercise, this group developed actionable guiding principles to achieve health and racial equity in cancer prevention and control research,[3] that are presented below.

Principle 1 (P1). Engage in power-sharing and capacity building with partners
Principle 2 (P2). Address community priorities through community engagement and co-creation
Principle 3 (P3). Explore and address the systems and structural root causes of cancer disparities
Principle 4 (P4). Build a system of accountability between research and community partners
Principle 5 (P5). Establish transparent relationships with community partners
Principle 6 (P6). Prioritize the sustainability of research benefits for community partners
Principle 7 (P7). Center racial equity in cancer prevention and control research
Principle 8 (P8). Engage in equitable data collection, analysis, interpretation, and dissemination practices
Principle 9 (P9). Integrate knowledge translation, implementation, and dissemination into research plans

[1] White A, Sabatino SA, Vinson C, Chambers D, White MC. The Cancer Prevention and Control Research Network (CPCRN): Advancing public health and implementation science. Prev Med. 2019 Dec;129S:105824. doi: 10.1016/j.ypmed.2019.105824. Epub 2019 Aug 29. PMID: 31473220; PMCID: PMC7032049.

[2] More information about the participating centers here: https://cpcrn.org/

[3] Chebli P, Adsul P, Kranick J, Rohweder CL, Risendal BC, Bilenduke E, Williams R, Wheeler S, Kwon SC, Trinh-Shevrin C. Principles to Operationalize Equity in Cancer Research and Health Outcomes: Lessons learned from the Cancer Prevention and Control Research Network. Cancer Causes and Control. 2023

Principle 1: Engage in power-sharing and capacity building with partners

Operationalizing this principle for research collaborations:

- Convene community advisory boards, committees or councils that can influence decision making around what research is proposed and funded
- Engage in transparent, deliberative processes to include and prioritize partners' voices into the academic research
- Use consensus building activities with partners to determine priorities for research
- Identify locally relevant evidence-based interventions
- Build capacity within the partnerships to engage in academic research including compensating partners for their contribution to the research project; involving partners in grant writing and manuscript development; disseminating research at conferences with partners as co-presenters and including support for travel to conferences
- Develop pathways for partners to lead research or practice-based projects and support learning exchanges and trainings, to cultivate research skills; and providing trainings and technical assistance to support development of research skills
- Evaluate the partnership and receive feedback about the collaboration from all perspectives

Practices undertaken or in-progress in CPCRN projects:

- Investigators have compensated partners, especially the community partners, for time spent on research projects
- Engaged in regular meetings between partners, with each partner participating equally in the conversations and decision-making for funded research, with respect to the partners preferences on where, how, and frequency with which to meet and interact
- Engaged in joint decision making with partners that are involved in the research process
- Actively engaged in consensus building activities with partners
- Engaged in community based participatory research-driven solutions and informed practices
- Incorporated mini-grant opportunities for community partners into research budgets
- Engaged in system-level implementation research in close partnership with existing community health care organization
- Lead community-engaged research training program for community partners to build capacity for research
- Used memoranda of understanding between partners

- Had a Community Advisory Board (CAB) to guide and inform decisions on research projects ranging from project conception to dissemination of findings

Reflection questions to guide researchers

- How are we assessing the priorities of the partners impacted by research?
- How can research and academic expertise serve the priorities for the partners?
- Are members of the practice community included on the research team?
- How are partners, especially community members, compensated for their participation in the research? Is the compensation aligned with the partner's needs and priorities?
- How are partnership power dynamics defined, acknowledged, and addressed?
- How are the partners engaged in joint decision making throughout the research process? Are partners engaged in shared decision making for research?
- What are some trainings and resources made available and accessible to partners that may not be familiar with research?

Resources and methods for assessment:

- The Power Mapping Tool helps assess which people or groups influence the decision maker and support your issue, what they think, what communities they identify with, and whether they are subject to competing views or priorities. This assessment provides a visual representation of where power relationships stand and what additional information from the broader social, political, and economic environment may be helpful.
- The Patient Centered Outcomes Research Institute (PCORI) provides a set of tools and resources to ensure engagement of partners, especially patients, in the research process
- Heller, J. et al., 2014, while suggesting the equity metrics for health impact assessment, suggests considering whether there is a shift in power to benefit communities, both within institutions and among communities when evaluating evidence (i.e., community data or knowledge as "expert" and valid evidence). Authors provide examples of approaches to shifting power, such as engaging in joining decision making with Community Advisory Boards
- Morrow, E., et al., 2010, provides a model measure to assess personal factors (one's ability, potential, and sense of being) as it contributes to their role in research collaborations.
- Popay, J., et al., 2021, in a three part series, discuss the importance of community empowerment as a strategy to achieve health equity. They propose two complementary frameworks (Emancipatory and Limiting Power framework) to shift Community empowerment approaches in the health field towards health equity.

Principle 2: Address community priorities through engagement and co-creation

Operationalizing this principle for research collaborations:

- Jointly develop, conduct, and analyze needs assessments in partnership with community members to identify shared goals and objectives
- Engage community partners in research design, implementation, analysis, and dissemination of research findings
- Integrate community input into recruitment and retention strategies to ensure equitable representation in enrollment and findings
- Convene and compensate community partners to provide oversight and guidance throughout the research process
- Prioritize research projects that are not extractive but rather in support of community priorities
- Critically examine the quality of community engagement strategies on whether communities are engaged throughout the research process
- Ensure community input is integrated into research products (i.e., grant applications, measures, dissemination)

Practices that have been undertaken in CPCRN projects:

- Included where possible, community partners as co-investigators on the grant
- Conducted research that stems from and is driven by community-defined needs and priorities
- Carrying out community needs assessments in partnership with community networks (e.g., design of instruments, recruitment for inclusion, etc.)
- Established formalized community partnerships (either a community advisory board or creating partnership agreements) to guide research process
- Co-created linguistically and culturally tailored study and intervention materials with community partners
- Investigators have engaged community members in research processes, including serving as co-authors and contributors on papers

Reflection questions to guide researchers:

- What efforts are made from the research team to identify and understand the community priorities and needs?
- What infrastructures and mechanisms are in place or can be leveraged at the research institution to engage and sustain community partnerships?

- How are communities being defined in the research? Do we have existing partnerships with entities that represent these communities?
- What funding resources may be leveraged to engage and sustain community engagement in research?
- Have we investigated whether the community considers the research being conducted valuable? Does the community believe that the proposed research benefits them?
- What are the motivations for the research team in conducting this research with this community?
- Has the research team critically examined their intentions in conducting the proposed research with the communities?
- Has the research team been trained in community engagement?
- How are voices from the communities and individuals with lived experiences around the health condition, included in the development of the research project?
- What are some opportunities created by the research team to gather voices from the communities and individuals with lived experiences around the health condition?
- How are community members recognized for their partnership in this process (e.g., presentation, reports, publications, etc.)?

Resources and methods for assessment:

- Conceptualizing community based participatory research:
  - Wallerstein, N. and Duran, B., 2010, presents the conceptual model of Community-Based participatory research and provides a rationale for including a community focus when considering interventions and implementation.
  - Goodman, M. and Thompson, V., 2017, discuss the key elements of implementation and evaluation of stakeholder engagement in research
  - Key, K, et al, 2019, present the continuum for community engagement which integrates a focus on health equity and contextual factors.
- Tools and resources for conducting community engaged research
  - The Community Toolbox provides resources and training for assessing community needs, addressing social determinants of health, engaging stakeholders, action planning, building leadership, improving cultural competency, planning an evaluation, and sustaining intervention/program efforts (More information here: https://ctb.ku.edu/en)
  - The Engage for Equity website (https://engageforequity.org/) provides access to the Community Based Participatory Research Model and the tools and resources that could be used in community engaged research projects.
  - Duea, s.et al, 2022, provides an overview for selecting the participatory research methods based on project and partnership goals.

- The University of Colorado's Dissemination, Implementation, and Community Engagement Guide provides a database of strategies for stakeholder engagement and education around assessment and application of these strategies
- Shea, C., et al., 2017., describe the core competencies for researchers to conduct community-engaged dissemination and implementation science

- Evaluating community engaged research -
  - Goodman, M., et al., 2016, presents the review of a community engagement measure consisting of 96-items. Later, Goodman, M., et al., 2021, also presents the development and validation of a brief Research Engagement Survey Tool (REST).
  - Bowen, D., et al., 2017, present a systematic review of measures assessing stakeholder engagement.
  - Luger, T., et al, 2020, present a systematic review of measures of community-engaged research, including measures related to context, process, and outcomes/impact.
  - Boursaw, B., el. al., 2021, presents the psychometric properties of survey instrument that helps assess the commitment to collective empowerment, community engagement in research activities, synergy, partner and partnership transformation, and projected outcomes

Principle 3: Explore and address the systems and structural root causes of cancer disparities

Operationalizing this principle for research collaborations:

- Develop a foundational understanding of the history and current realities of social and structural issues
- Ground research projects in critical race, socio ecological, and life course approaches and paradigms (e.g., transformative, emancipatory) to health, moving beyond individual and interpersonal levels of influence, with a focus on addressing organizational, neighborhood, systems, and/or policy level causes of cancer disparities
- Collaborate with partners that understand the root causes of inequities and work together to design or select interventions to address them
- Use theory-based, partnership-guided processes such as implementation and intervention mapping, to design or select interventions and strategies that can address systems and structural level causes of inequities
- Engage partners that can influence multiple socio-ecological levels and represent multiple different sectors beyond the health sector
- Work to understand how racism as a social and structural determinant of health creates inequities (i.e., education, healthcare access, economic stability, built environment, among others) and incorporate a focus on addressing racism
- Leverage quantitative data sources to capture complex social phenomena around racism
- Use qualitative data sources to co-creating and generate new knowledge that amplifies historically excluded voices, perspectives and experiences related to structural and root causes of inequities

Practices that have been undertaken in CPCRN projects:

- Engaged community partners in identifying root causes of health conditions
- Identified root causes of inequities and potential means to address them
- Used implementation mapping to identify and address structural and environmental causes of health conditions
- Expanded knowledge and skills in addressing racism and health equity through participation in trainings provided by the Racial Equity Institute
- Implemented multi-level interventions that simultaneously address barriers to healthcare access from patient, provider, systems, community, and/or policy levels

Reflection questions to guide researchers:

- Is your proposed research incorporating a focus on upstream factors that truly address and impact social determinants of health?
- How are you addressing multiple socio ecological levels of influence in your proposed research?
- How does your proposed research assess the implications in terms of policy and practice?
- How are you integrating a focus on or addressing structural determinants in your research?
- How do the theories, models, or framework used in your research, conceptualize social and structural determinants of health?
- How is context considered in your research project? What formative assessments can help you understand community context? Are these assessments focused on identifying and understanding social and structural determinants of health?
- How are you measuring the impact of structural determinants of health in your proposed research?
- Are your interventions or implementation strategies addressing structural determinants to improve access to evidence-based interventions?
- How are you translating research findings to influence policy-level changes that address the social determinants of health?
- How are you disseminating findings/making connections with policy makers to make a larger impact on policy changes?
- What is the role of leadership/co-leadership or diverse representation among leadership of research teams?

Resources and methods for assessment:

- Conceptualizing multilevel influences on health -
  - National Institute on Minority Health and Health Disparities (NIMHD) Research Framework. Presents factors in determining the different domains and levels of influence to understand and address the causes of health disparities.
  - Taplin, S., et al., 2012, provide a multilevel model to consider the influence on healthcare delivery.
  - Zahnd, W., et al., 2019, provide a multilevel conceptual framework that describes how rural residence and relevant micro, macro, and supra-macro factors can be considered in evaluating disparities across the cancer control continuum.

- Multilevel Intervention Training Institute (MLTI). Application based training program on multilevel intervention research (MLI) provided by The National Cancer Society (NCI) Division of Cancer Control and Population Sciences (DCCPS)

- Measuring and conceptualizing structural racism -
  - Bailey, Z., et al, 2017, provide a conceptual report on the contemporary and historical perspectives to discuss research and interventions around structural racism and its impact on population health and health disparities.
  - Robinson, W., et al., 2020, provide a commentary on the importance of considering structural racism in machine learning models and thereby improving their accuracy.
  - Shelton, R., et al., 2021, provide a call to action in the field of implementation science, encouraging a specific focus on structural racism.
  - Hardeman, R., et al,, 2022, provide a review of research on structural racism, identify data sources and measures to evaluate the impact on health and healthcare.
  - Dean, L, et al., 2022, provide a commentary on the definition of structural racism and different approaches to evaluating its impact on health.
  - Adkins-Jackson, P., et al., 2022, provide a commentary on the methodological and analytic recommendations for measuring structural racism.

- Incorporate multi-level determinants/factors into data collection, analysis, and interpretation, to measure relevant factors influencing cancer.
  - PhenX Social Determinants of Health Assessments Collection, by the National Institute of Minority Health and Disparities, presents a collection of variables to capture the social determinants of health, including structural determinants such as poverty and neighborhood segregation.
  - HDPulse Data Portal,by the National Institute of Minority Health and Disparities, provides county-level data for characterizing the burden of disparities and examining determinants along a social ecological framework.
  - Minority Health Social Vulnerability Index, by the Centers for Disease Control and Prevention, provides county-level data to examine social vulnerability.
  - The GIS Portal for Cancer Research, by the National Cancer Institute provides a web resource for visualizing and mapping cancer outcomes data.
  - Build Health Equity with Data Science | RTI Tech Talk Webinar, provides a review of the development and utility of Local Social Inequity Score (LSI).
  - Places. Local Data for Better Health is a web resource that provides health measures across the US according to geography at the county, place (incorporated and census designated places), census tract, and ZIP Code Tabulation Area (ZCTA).

    - Zahnd, W., et al., 2017, provide a systematic review of studies using multi-level modeling in relation to cancer outcomes

- Use Implementation/Intervention Mapping framework to identify and address structural and environmental level causes of health problems.
    - Fernandez, M., et al., 2019, provides a review of the six-step intervention mapping process which incorporates multi-level factors that affect health.
    - Fernandez, M, et al, 2019, describes Implementation Mapping, an expansion of step 5 in Intervention Mapping (Step 5: “Plan for adoption, implementation, and sustainability of the program in real-life contexts by identifying program users and supporters and determining what their needs are and how these should be fulfilled.”)

Principle 4. Build a system of accountability between research partners

Operationalizing this principle for research collaborations:

- Understand and document the origins of the research partnerships
- Discuss and formalize goals for researchers and partners involved in research
- Create standard operating procedures to explicitly incorporate a focus on collaborative work, with each partners goal identified and addressed
- Collect metrics and narrative descriptions of partnership progress via routine reporting
- Re-visit and assess change in goals and partnership objectives periodically throughout partnership
- Develop or select the use of context-appropriate measures to assess impact of research on social, systems, and structural determinants of health and inequities
- Report sub-population specific findings to community partners and to the research community
- Assess, with statistical rigor, both implementation and effectiveness outcomes across sub-populations experiencing disproportionate burden of disease (e.g., by socioeconomic status, racial/ethnic populations, rural/urban populations)
- Explicitly focus on documenting partnership assets for research

Practices that have been undertaken in CPCRN projects:

- Assessing the distribution of both implementation and effectiveness outcomes across racial/ethnic, rural/urban populations in most of our projects
- Including a focus on access to cancer treatment, in addition to cancer prevention
- Co-developing or co-selecting research measures with partners
- Giving mini-grants to community partners thereby providing more resources to use existing data and collect new where needed.

Reflection questions to guide researchers:

- What are some ways in which we as researchers can improve accountability towards community partners?
- How are we documenting each partner's goals and objectives for their involvement in the research partnership?

- How does a researcher move beyond the competitive nature of traditional academic research towards collaborative research?
- How do my community partners evaluate our partnership / me / our science?
- How are we building a systematic process to disseminate findings back to partners?

Resources and methods for assessment:

- The River of Life exercise, within the tools of Engage for Equity from the Center for Participatory Research at the University of New Mexico, is a reflective tool that helps partnerships describe their history and document critical moments of the partnerships journey.
- The Green Book of Community Development, provides asset-based approaches to community development. They provide a toolkit, which reminds researchers to not just look at deficits but capacity and strengths in communities.
- The William T Grant foundation's Assessment of Research Partnership's provides guidance on the five dimensions by which research-academic partnerships can be effective, including indicators for measuring these in partnership processes
- Hoekstra, F., et al., 2018, provide an evaluation of research partnerships using the Reach, Effectiveness, Adoption, Implementation, and Maintenance (REAIM) framework
- VanDevanters, N., et al., 2013, provide an evaluation of community-academic partnership functions, using mixed methods
- London, et al. 2022, provide community partner perspectives on academic-research partnerships. They also discuss important considerations towards equitable collaborations, in a recent blog post, titled, "Toward equitable collaboration: Community partners' strategic perspectives on community-engaged research"
- Schaal, J., et al., 2016, presents focus group data on the collaborative processes of the community-academic partnership guiding the Accountability for Cancer Care through Undoing Racism and Equity (ACCURE) study that is examining the impact of a systems-change intervention aimed at addressing disparities.

Principle 5. Establish transparent relationships with research partners

Operationalizing this principle for research collaborations:

- Hold space for discussions around power dynamics between research partners
- Establish co-defined, roles and expectations (both formal and informal) with partners
- Share and discuss project updates and research progress in the agreed upon frequency, including outcomes related to cancer disparities and racial equity in plain language briefs developed in partnership
- Work with research partners to interpret study findings and their implications
- Incorporate regular check-ins (either as a group or separately to minimize power dynamics) to ensure that partners are achieving their own objectives for project participation and discuss strategies to address, if not being achieved
- Invest time in developing relationships with the community partners to foster a culture of trust and avoid perpetuating the historically extractive nature of research
- Increase familiarity of the academic fiscal and administrative processes and financial management expectations among partners, which is often overlooked but is important for increased transparency.
- Adopt a learning-centered approach to shift priorities in research towards more collaborative active learning when conducting and collaborating on projects
- Consider the use of open science avenues to share and improve research performance

Practices that have been undertaken in CPCRN projects:

- Creating memoranda of understanding with research partners that outline expectations of each partner in the research
- Establishing relationships and setting expectations at the beginning of the relationship
- Developing briefs to report project results to partners.
- Ensuring that every step of collaborative work is relational with shared responsibilities and expectations.
- Meeting regularly and discussing roles and interests of partners at each stage of research.

- Meeting and reporting regularly to partners in activities. Being mindful of collecting data that will be publicly shared with all partners and funders as a reflection of the services we provide to and impact upon the larger community.

Reflection questions to guide researchers:

- Have roles and responsibilities been clearly defined? If so, how were the roles and responsibilities created and discussed?
- Do the research partners have the opportunity to shape the direction of the research projects in how it impacts their work and goals? If so, how often?
- Do research partners get a chance to set the agenda or to present aspects of the work that are most meaningful to them?
- Have research partners been provided with the resources they need to support the project?

Resources and methods for assessment:

- Yuan, N., et al, 2020, describes the development of guidelines for community advisory boards. They are also conducting an ongoing study to evaluate the effectiveness of these guidelines.
- Newman, S., et al, 2011, describes best practices for developing, operating and maintaining community advisory boards.
- Cramer, M., et al., 2018, present a case study conducted with members of a rural community advisory board; their perspectives on opportunities and challenges of working with academic partners are presented.
- James, S., et al., 2011, describes a model to depict how a community advisory board prioritized action and relationship building to increase diversity, participation, transparency, mutual respect and recognition.
- Emmons, K., et al, 2022, describes the Community Coalition for Equity in Research, which is a community-driven resource designed to increase two-way engagement between researchers and the larger community. Additional details around the community coalition are presented on their webpage.
- Urban Institute's 2021 Tools and Resources for Project-Based Community Advisory Boards, provides a toolkit for practical guidance, questions, and approaches for incorporating community advisory Boards into a project.
- The Southern California, Clinical and Translational Science Institute, provides a toolkit for developing a community advisory board for research
- The Community Campus Partnerships for Health provides several resources for equitable partnerships

- Examples for Memoranda of Understanding for Community Partners:
    - The University of California Los Angeles Clinical Translational Science Institute's community engagement office provides guidance
    - The University of Kentucky provides a template for the Memorandum of Agreement
    - Additional examples provided at the Community Partnerships Knowledge Hub
    - Community Camp

- The National Institutes of Health's Certificate of Confidentiality (CoC) can help protect the privacy of the research participants by prohibiting the disclosure of identifiable, sensitive research information to anyone outside of the research team.
- Matenga, T., et al., 2021, presents findings from a qualitative study with Zambian researchers to understand their perspectives on authentic partnerships with Southern and Northern partners.
- Skewes, M., et al., 2020, presents the methodological process of how researchers worked with American Indian and Alaska Native individuals to develop and conduct survey research focused on substance use.
- Akinremi, T., 2011, presents a review of literature about challenges and solutions of high resource countries collaborating with low resource countries to conduct research.

Principle 6. Prioritize the sustainability of research benefits for community partners

Operationalizing this principle for research collaborations:

- Conduct an environmental scan with partners to gauge landscape of current health system and policies that might help or hinder the intervention
- Align interventions proposed for research with existing structures/capacity in mind (e.g., practice-based research network, health systems, organizational and systems change)
- Assess sustainability goals and objectives from community and academic perspectives, including mutual understanding of resources required and opportunity costs
- Develop interventions in collaboration with partners to ensure sustainable continuity of outcomes
- Incorporate opportunities to train and build infrastructure (e.g., trained community health workers), for long-term continuity of proposed intervention/research
- Apply an ethical lens to building long standing relationships as a continued resource for public health planning within the community (e.g., continue to serve as technical assistance resource for community partners past grant period)
- Integrate partners from multiple interpersonal and inter-organizational levels so that the partnership is not dependent on a single partner

Practices that have been undertaken in CPCRN projects:

- Developing long-standing relationships with partners and regularly respond to partner requests for support and collaboration
- Working towards increasing funding for partners by engaging in planning, response to grants, provide letters of support, and share resources, and expertise
- Grounding interventions in current systems or practice based research
- Building sustainability from the start of the research project

Reflection questions to guide researchers:

- What resources exist to sustain proposed intervention after the end of grant funding?
- Who/what are the needed components to ensure sustainability of intervention?
- Can the research team obtain funds to either sustain or create these components through grant funding? If I cannot, is it ethical to conduct this research?
- How can research findings support long-term partners' goals?

Resources and methods for assessment:

- The Program Sustainability Assessment Tool can be used to rate the sustainability of the program to help for its future
- Coombe, C., et al, 2020, describes the process to develop a validated instrument to measure success in long-standing community based participatory research partnerships
- Arora, P., et al, 2015., came up with a measure to characterize the range of relationships between researchers and community members engaging in community-based research
- Using sustainability as a collaboration magnet to encourage multi-sector collaborations for health: https://pubmed.ncbi.nlm.nih.gov/28353396/
- Hall, T., et al, 2021, describes the facilitators and barriers to the sustainability of research partnerships
- Shelton, R., et al., 2018, provide an in-depth review around the sustainability of evidence-based interventions in public health and healthcare

Principle 7. Center racial equity in cancer prevention and control research

Operationalizing this principle for research collaborations:

- Consider the historical and contextual implications of racism throughout the research process
- Reflect on individual and research team's inherent and unconscious racial biases and evaluate implications on proposed research
- Support research team member's continuous training and education on the role of systemic racism and discrimination on health
- Integrate diversity in research teams to reflect communities being served through the research
- Support recruitment and retention strategies of underrepresented students and partners in research teams
- Prioritize personal or individual-level efforts towards educating oneself regarding the historical impacts of racism on impacted partner communities
- Include structural racism and health equity methodological experts in research teams
- Integrate anti-racist approaches to development and implementation of intervention strategies and in the collection of survey and data measures
- De-center majority perspectives by shifting a focus and intentionality on examining 'otherness' and 'marginalized social positions' in research
- Utilize Indigenous research methods and decolonizing research practices
- Formalize racial equity work into training plans for trainees and researchers.
- Incorporate racial equity work into job duties for research staff.
- Use non-stigmatizing language to describe populations in research

Practices that have been undertaken in CPCRN projects:

- Providing and supporting training in bias as well as integrating diversity in research teams
- Adding racial equity issues as a consistent item on agenda sharing materials, programs, and information, to advance understanding.
- Committing staff time to participate in additional training in diversity, equity and inclusion approaches
- Integrating racial equity as an integral component and committing time for individuals in the research team to engage in "inner work," where we are supposed to be challenging ourselves and growing

Reflection questions to guide researchers:

- Are you engaged in an ongoing process of explicitly examining and addressing implicit biases?
- Is the research addressing community concerns around racial inequities?
- Do you understand community concerns around health disparities and racial equity? What information may be missing?
- Who have you talked with from the community in gathering this information? Who is missing from this conversation?
- Is racial equity incorporated into research trainees' training plans?
- Can we as the research team do more to educate ourselves on implicit bias, and diverse experiences and perspectives?
- Are we placing the onus on community partners or members of the community we collaborate with to educate us on race and experiences of racism as applicable to the research context? Can we do more to educate ourselves before we enter these community spaces?
- Is your research team trained in understanding the nuances of unconscious bias and how it influences the way they engage with study participants or community partners?

Resources and methods for assessment:

- Shelton, R. C., et al, 2021, call for an anti-racism lens in the science of dissemination and implementation
- VanderWeele & Robinson, 2014, discuss the several possible interpretations when considering the effect of race when running regression model and adjusting for confounding and mediating variables
- Howe, C., et al., 2022, discuss the use of causal diagram to study racial health disparities
- Ward, J,. et al, 2019, describe a comprehensive framework for studying racial disparities, with a focus on examining group-specific difference in outcome prevalence, exposure prevalence, and effect size.
- Raque, T., et al, 2021, describe the Multicultural Orientation Framework that can guide health psychologists to consider the sociocultural and political history of their work, systems of oppression and privilege embedded in health research, and a path toward using research to achieve social change, antiracism, and health equity.
- Boyd, R., 2020., describes standard for publishing on racial health inequities
- The Institute of Healing and Justice in Medicine provides an important summary to deconstruct race as a biological construct
- The Centers for Disease Control and Prevention provides the Preferred Terms for Select Population Groups & Communities which describes the preferred terms for various populations and communities

Principle 8. Engage in equitable data collection, analysis, interpretation, and dissemination practices

Operationalizing this principle for research collaborations:

- Consult with community partners to refine which health outcomes and/or social/structural determinants to measure
- Choose, collect and analyze measures that reflect adherence to the health and racial equity principles herein
- Evaluate to the full extent the need and relevance of each data collection measure
- Identify and plan in advance the purpose of each data collection measure
- Establish co-ownership of data with partners through data sharing agreements and involve partners in all stages of data collection, analysis, interpretation, and dissemination
- Collect and analyze disaggregated data by race/ethnicity and by subpopulations
- Identify and utilize appropriate data collection tools and techniques that have been validated for the intended population
- Report back collected data to the research partners in a timely manner
- Consult with research partners to identify ideal method of report back of findings (e.g., email or electronic methods may not always be appropriate)

Practices that have been undertaken in CPCRN projects:

- Implementing data collection protocols that reduce partnership burden as much as possible, e.g. ask partners if/when they have existing institutional/state/federal reporting requirements and deadlines, then time research data collection around partners' other reporting deadlines. For example, FQHCs' required reports to HRSA are usually due Jan/Feb of each year, so we avoid asking them for any data leading up to/during this time.
- Engaging community partners and stakeholders in the dissemination of scientific findings such as formal and informal presentations to the community and CABs
- Developing a data use agreement template for community partners to ensure researchers are ethically using the data and complete transparency in the use of the data moving forward
- Actively engaging community partners from the early days, disseminating all outcomes/ metrics as they evolved and gaining community input on what to measure, share and how to best share.
- Engaging community partners in qualitative data interpretation and plans for reporting

Reflection questions to guide researchers:

- Are the tools and data collection methodologies capturing the exposures and outcomes to the full extent in the population of interest?
- Are the tools and data collection methodologies adequate and appropriate for the population that they are been implemented on?
- Do community partners and stakeholders agree/ approve on the adequacy of assessment tools being implemented?
- Do community partners and community members agree on the interpretation and contextualization of the data?
- Is the data being collected relevant and needed to answer the proposed research question?
- Is there a true balance between the imposed participant burden for data collection and purpose of the data being collected?
- How is the data going to be disseminated in an equitable manner for the greatest reach and benefit of the community?

Resources and methods for assessment:

- The Data Equity Framework, provides a systematic approach, through a set of tools, checklists, and practices, to help researchers make intentional project choices that facilitate attainment of their equity goals and priorities.
- The CARE (Collective benefit, authority to control, responsibility, and ethics) principles for Indigenous Data Governance, are a way to position data approaches within Indigenous cultures and knowledge systems to the benefit of Indigenous peoples and honor the FAIR guiding principles for scientific data management and stewardship.
- The Urban Indian Health Institute's website includes data dashboards, urban Indigenous organization and community profiles, and links to specific research projects.
- Stella Yi, et al, 2022, describe the mutually reinforcing cycle of health disparities among Asian American, due to poor-quality data infrastructure and biases on the part of researchers and public health professionals, while providing recommendations on how to infuse racial equity in future policy and practice.
- Hilliard-Boone, T., et al, 2022, provides guiding principles for measurement in equitable outcomes
- Lett, E., et al., 2022, outlines pitfalls in the conceptualization, contextualization, and operationalization of race in quantitative population health research
- Dover & Belone, 2019, provide a partnership driven, conceptual frameworks to measure the impact of social determinants of health through causal pathways
- Flanagin, A. et al, 2021, provide updated guidance on reporting of race and ethnicity in medical journals

Principle 9. Integrate knowledge translation, implementation, and dissemination into research plans

Operationalizing this principle for research collaborations:

- Co-create solutions with practice-oriented partners to facilitate translation of evidence-based research and policy into effective community and clinical practice
- Integrate dissemination through trusted, community-focused venues in project and engagement goals
- Tailor dissemination strategies to partners' goals, values, literacy, language, and cultural needs
- Disseminate and adapt findings to diverse audiences (e.g., policymakers, oncology care providers, primary care providers, community members)
- Select implementation strategies that emphasize equitable reach across diverse communities

Practices that have been undertaken in CPCRN projects:

- Involvement of practitioners has meant that research projects have implications for implementation in practice environments
- Development of coalitions, plans, policies and practices together contribute to knowledge translation
- Making sure that dissemination, implementation and scalability are incorporated into research plans with sufficient resources

Reflection questions to guide researchers:

- Are we involving the relevant policy makers and practitioners in informing the development of the research project?
- Have we involved diverse dissemination partners in the research project?
- Who are we considering as dissemination partners?
- What dissemination tools are optimal for different audiences (e.g., newsletters, policy briefs, infographics, etc.)?
- Do we have a sustainability plan in place to ensure components of projects that are beneficial to partners can be sustained?

Resources and methods for assessment:

- The Centers for Disease Control and Prevention has a Sustainability Planning Guide that helps researchers with science- and practice-guided evidence to develop, implement, and evaluate a successful sustainability plan. (More information here:

https://www.cdc.gov/nccdphp/dch/programs/healthycommunitiesprogram/pdf/sustainability_guide.pdf)

- The Center for Public Health Systems Science at the Brown School at Washington University in St. Louis developed the Program Sustainability Framework and Assessment Tool (PSAT) to Understand, Assess and Plan for Program Sustainability. (More information here: https://www.sustaintool.org/psat/about-us/)
- The Designing for Dissemination Toolkit developed by the University of Colorado provides researchers with the scientific rationale for designing for dissemination, helps identify the key processes, outcomes and products for designing, and outlines a plan for design, evaluation, and dissemination of research products that takes end users into account. (More information here: https://medschool.cuanschutz.edu/docs/librariesprovider94/di-docs/guides-and-tools/2018-d4d-workbook_revised2.pdf?sfvrsn=463c06b9_2)
- The Translational Science Benefits Model allows researchers and research institutions to consider the broader impacts of research, on clinical and community health impacts, beyond bibliometrics and grant funding. (Luke, D., et al., 2017)
- The Sci Comm Toolkit, developed by the Society of Behavioral Medicine, provides resources needed to communicate science effectively, thereby helping share science with the public. (More information here: https://www.sbm.org/scicomm)
- The resource Implementation Science at a Glance, developed by the National Cancer Institute helps practitioners and policy makers gain familiarity with the building blocks of implementation science. (More information here: https://cancercontrol.cancer.gov/sites/default/files/2020-07/NCI-ISaaG-Workbook.pdf)
- The Patient Centered Outcomes Research Institute has a Dissemination and Implementation Framework and a toolkit to help researchers facilitate strategic planning to increase the awareness of evidence and promote its integration into practice. (More information here: https://www.pcori.org/impact/putting-evidence-work/dissemination-and-implementation-framework-and-toolkit)
- The National Implementation Research Network's Active Implementation Hub: Provides a free, online learning environment for researchers and practitioners active in implementation and scaling up of programs and innovations. (More information here: https://nirn.fpg.unc.edu/ai-hub)

Overarching Bibliography and Resources:

- The Equity Journey Training Program, provides a three-module, self-guided approach to understanding equity and bias principles and structural and system-level barriers, providing strategies to align professional practice with social equity objectives, and creating an action plan.
- NACCHO's Health Equity and Social Justice Toolkit is a searchable database of health equity tools, publications, and resources available through the National Association of County and City Health Officials (NAACHO) toolbox.
- The Colorado School of Public Health has curated list of available trainings and resources around health equity. In addition, they also host a Stakeholder Engagement Navigator tools and guides for research interested in Dissemination, Implementation, Communication and Engagement
- The Centers for Disease Control and Prevention, also provide a list of resources around Health Equity, in addition to a focused guide on practitioners: A Practitioners Guide for Advancing Health Equity: Community Strategies for Preventing Chronic Disease
- The Washington University in St Louis, provides a Community-Engaged Research and Practices' Resource Site, in partnership with the Bernard Becker Medical Library and the Center for Community Health Partnership and Research
- The University of New Mexico's Center for Participatory Research provides access to several tools from the Engage for Equity study of community-academic partnership
- The National Cancer Institute through the Cancer Consortium for Implementation Science provides critical bibliography and resources in Advancing Health Equity Through Implementation Science: Bibliography and Resources. In addition, there is a separate bibliography focused on Community engagement.
- A series of webinars by the National Institute of Minority Health and Health Disparities, provides discussion around structural racism and health equity
- The Dimensionality Framework (Hogan, V., et al, 2018) provides operationalization of the equity framework into actionable steps
- Luger, T., et al, 2020 provide a mapping review of measuring context, processes, and outcomes
- The National Academies of Science and Engineering and Medicine, recently proposed the Pathways to Health Equity Model with supporting measurement
- The National Academies of Science and Engineering and Medicine, recently released guidance on the standardize measurement around Gender and Sexual Orientation
- Understanding catchment areas for the National Cancer Institute's Community Outreach and Engagement Offices -

- Catchment Areas and Community Outreach and Engagement: The New Mandate for NCI-Designated Cancer Centers
- A National Map of NCI-Designated Cancer Center Catchment Areas on the 50th Anniversary of the Cancer Centers Program

- A three-part series highlights learnings from Lead Local: Community-Driven Change and the Power of Collective Action, a collaborative effort funded by the Robert Wood Johnson Foundation:
  - Building Community Power to Achieve Health and Racial Equity: Principles to Guide Transformative Partnerships with Local Communities
  - Community Power and Health Equity: Closing the Gap between Scholarship and Practicehttps://nam.edu/wp-content/uploads/2022/06/Community-Power-and-Health-Equity_FINAL.pdf
  - Why Community Power Is Fundamental to Advancing Racial and Health Equity

- Gollust, et al,, 2022, review of the Interdisciplinary Research Leaders (IRL) program curriculum from the Robert Wood Johnson Foundation. The goal of the curriculum is to educate researchers in advancing health equity.
- Behar-Horenstein, I., et al., 2021, provide measurement instruments for the common evaluation metrics for the Comprehensive Partnerships to Advance Cancer Health Equity (CPACHE) Program which may have utility in other contexts.

- Wilf-Miron, R., et al., 2021, provide the process used to develop a national set of health equity indicators, and present those indicators. Researchers might find the process informative, as well as the indicators developed.

- Textbooks for community engagement research:
  - Community-Based Participatory Research for Health: Advancing Social and Health Equity, 3rd Edition
  - Principles of Community Engagement, 2nd Edition

CPCRN
Cancer Prevention and Control Research Network

Acknowledgements

We sincerely thank the contributors listed below for their insights into the development of this toolkit.

Hannah Arem, PhD; MedStar Health Research Institute
Enmanuel Chavarria, PhD; Emory University
Perla Chebli, PhD, MPH; NYU Grossman School of Medicine
Jean Edward, PhD, RN; University of Kentucky
Cam Escoffery, PhD, MPH, CHES; Emory University
Tisha Felder, PhD, MSW; University of South Carolina
Victoria Foster, MPH; NYU Grossman School of Medicine
Rachel Hirschey, PhD, RN; University of North Carolina at Chapel Hill
Jessica Islam, PhD, MPH; Moffitt Cancer Center
Julie Kranick, MPhil, MA; NYU Grossman School of Medicine
Simona Kwon, DrPH, MPH; NYU Grossman School of Medicine
Melissa Lopez-Pentecost, PhD, RDN; University of Miami
Sarah Nash, PhD, MPH, CPH; University of Iowa
Antoinette Percy-Laurry, DrPH, MSPH; National Institute on Minority Health and Health Disparities
Rashmi Sharma, MD, MHS; University of Washington
Betsy C. Risendal, PhD; University of Colorado Cancer Center
Catherine L. Rohweder, DrPH; The University of North Carolina at Chapel Hill
Thuy Vu, MPH; University of Washington
Arica White, PhD, MPH; Centers for Disease Control and Prevention
Stephanie Wheeler, PhD, MPH; The University of North Carolina at Chapel Hill

(Health Equity Workgroup co-chairs)
Prajakta Adsul, PhD, MBBS, MPH; University of New Mexico Comprehensive Cancer Center
Chau Trinh-Shevrin, DrPH; NYU Grossman School of Medicine
